# Rheumatic heart disease in Uganda: predictors of morbidity and mortality one year after presentation

**DOI:** 10.1186/s12872-016-0451-8

**Published:** 2017-01-07

**Authors:** Emmy Okello, Chris T. Longenecker, Andrea Beaton, Moses R. Kamya, Peter Lwabi

**Affiliations:** 1 Uganda Heart Institute, Kampala, Uganda; 2 Department of Medicine, School of Medicine Makerere University, Kampala, Uganda; 3 Division of Cardiology, University Hospitals Harrington Heart and Vascular Institute, Case Western Reserve University School of Medicine, Cleveland, OH USA; 4 Department of Cardiology, Children’s National Medical Center, Washington, DC USA; 5 Uganda Heart Institute/Department of Medicine, Makerere University, First Floor Block C, Mulago Hospital Complex, PO Box 7051, Kampala, Uganda

**Keywords:** Rheumatic heart disease, Predictors, Morbidity, Mortality, Outcomes, Uganda

## Abstract

**Background:**

Rheumatic heart disease (RHD), the long-term consequence of rheumatic fever, accounts for most cardiovascular morbidity and mortality among young adults in developing countries. However, data on contemporary outcomes from resource constrained areas are limited.

**Methods:**

A prospective cohort study of participants aged 5–60 years with established RHD was conducted in Kampala, Uganda, in which clinical exam, echocardiography, electrocardiography (ECG), and laboratory evaluation were done every 3 months and every 4-week benzathine penicillin prophylaxis was prescribed. Participants were followed up for 12 months and outcomes and predictors of morbidity and mortality were assessed using Kaplan Meier curves and Cox proportional hazards models.

**Results:**

Of 449 subjects, 66.8% (300/449) were females, median age was 30 (interquartile range 20). 73.7% (331/449) had atleast one follow up visit. Among these, 35% (116/331) developed decompensated heart failure and, 63.7% (211/331) developed atrial fibrillation. Heart failure was associated with poor penicillin adherence (OR = 3.3, CI 2–5.4, *p* = 0.001), and left ventricular end diastolic diameter greater than 55 mm (OR = 3.16, CI 1.73–5.76, *p* = 0.001). Atrial fibrillation was associated with left atrial diameter >40 mm (OR = 7.5, CI 2.4–9.8, *p* = 0.001).

There were 59 deaths with a 1-year mortality rate of 17.8%. Most deaths occurred within the first three months of presentation. Subjects whose average adherence to benzathine penicillin was <80% had significantly greater mortality (31% vs. 9%, log rank *p* < 0.001). In multivariate analysis, the risk of death among those with poor penicillin adherence was 3.81 times higher than those with better adherence (HR = 3.81, CI 1.92–7.63, *p* = 0.001). Other predictors of 1 year mortality included heart failure (HR 8.36, CI 3.28–21.31, *p* = 0.001) and left ventricular end diastolic diameter greater than 55 mm (HR = 1.93, CI 1.07–3.49, *p* = 0.02).

**Conclusion:**

In this study of RHD in Uganda, morbidity and mortality within 1 year of presentation were higher than in recently published from other low and middle income countries. Suboptimal adherence to benzathine penicillin injections was associated with incident heart failure and mortality over 1 year. Future studies should test interventions to improve adherence among patients with advanced disease who are at the highest risk of mortality.

## Background

Rheumatic heart disease (RHD) remains a leading cause of morbidity and mortality among young adults in the developing world, accounting for at least 345,000 deaths annually [1, 2].

RHD is the long term consequence of rheumatic fever, an autoimmune response to Group A streptococcal pharyngitis [3].

Without prophylaxis, patients with RHD are at risk of recurrent attacks of rheumatic fever resulting in ongoing inflammation and fibrosis with consequent valvular damage [4, 5].

In Uganda, RHD is the most common cause of heart disease in young adults [6]. As is true in many underserved areas, Ugandan patients tend to present late in the disease course, and almost half present with a complication [7].

Longitudinal studies of acute rheumatic fever (ARF) and RHD suggest that adherence to penicillin prophylaxis can significantly decrease disease progression and mortality [5, 8, 9].

However, these data come from patients early in disease course, who were most often enrolled following diagnosis of ARF. Unfortunately, our patients tend to present much later with complicated RHD. Whether penicillin prophylaxis improves outcomes among patients with more advanced disease is less clear.

Additionally, the lack of good quality data on RHD progression and outcomes in these regions limits efforts to target appropriate disease prevention and treatment strategies in settings where cardiac surgery is not widely available. We therefore aimed to describe the incidence of morbidity and mortality among a cohort of Ugandan patients with RHD within the first year after diagnosis, and to determine predictors of these events.

## Methods

A prospective cohort study of patients with established RHD was conducted in Kampala, Uganda from 2011 to 2013. The study was conducted in parallel with recruitment into a global RHD registry (the REMEDY study) [10].

Subjects were co enrolled and underwent additional investigations as part of this study. Participants aged 5–60 years were enrolled at initial presentation and followed up quarterly for 12 months. Recruitment occurred from within the Mulago National Referral hospital including; the Uganda Heart Institute, the inpatient cardiology ward, and the outpatient cardiac clinics. Additionally, physicians from other Ugandan hospitals were invited to refer patients to the study site.

Participants were eligible for the study if they met World Heart Federation criteria for definite RHD by echocardiography. Participants who were unable to consent or who had morphological features of congenital or degenerative valve disease were excluded.

### Study procedures

At baseline, a detailed history was taken from consenting participants, after which they underwent physical examination, chest x-ray, 12-lead electrocardiogram, and venous blood draw for analysis of antistreptolysin O titers and C reactive protein. At each subsequent visit, interval history and exam were obtained, including symptoms of ARF, ECG, chest x-ray, and laboratory tests were repeated. All patients were prescribed 4 weekly intramuscular benzathine penicillin prophylaxis. RHD complications were treated according to local standards of care. Penicillin injections were recorded on a patient adherence card and the percentage adherence was determined each quarter by dividing the number of injections received by the number prescribed. Cumulative adherence was then averaged for each quarter over the 12 months. Adherence to >80% of prescribed injections is considered optimal.

### Echocardiography

A standardized echocardiogram was performed according to American Society of Echocardiography guidelines [11, 12] using a GE Vivid 7 (GE, USA). Two-dimensional and color images as well as M-mode were used to obtain left ventricular end diastolic diameter (LVDD), left ventricular end systolic diameter (LVSD), and ejection fraction (EF).

Mitral stenosis was determined by pressure half-time, mean gradient, continuity equation and/or planimetry of the valve orifice in the parasternal short axis view. Mitral stenosis was graded according to mitral valve area (MVA) as mild (1.6–2.0 cm^2^), moderate (1.1–1.5 cm^2^), or severe (<1 cm^2^). Mitral regurgitation was graded according to regurgitant jet area as mild (<4 cm^2^), moderate (4–8 cm^2^), or severe (>8 cm^2^). In addition, severe MR was diagnosed if there was flow reversal in the pulmonary veins. Aortic stenosis was determined by the calculated valve area by continuity equation and graded as mild (aortic valve area 1.6–2 cm^2^), moderate (1.1–1.5 cm^2^), or severe (<1 cm^2^). Aortic regurgitation was determined by pressure half time and graded as mild (PHT >500 ms), moderate (PHT = 250–500 ms) or severe (PHT <250 ms). Tricuspid and pulmonary valve involvement were rare and were not quantified for this study.

### Biomarkers

Six mililiters of venous blood was drawn at each visit to measure antistreptolysin O titer (ASO) and C reactive protein (CRP). ASO titers were estimated using the latex agglutination technique of immunoturbidimitry. (Cobas Integra 400, Roche, Germany). A conservative test value of 240 Todd units was taken as positive to cater for the variation in ASO titers for the different age ranges [13]. CRP was determined using the particle enhanced turbidimitry assay. (Cobas Integra 400, Roche, Germany).

### Outcomes measures

Outcome measures, assessed at each visit, included cardiac related death (determined based on death certificates), atrial fibrillation (Minnesota coding, [14] heart failure (Framingham [15]) and severity (NYHA classification), Stroke (clinical neurological deficit and confirmation with head CT), recurrent ARF (2006 NIH/WHO criteria) [16], and infective endocarditis (clinical presentation, blood cultures and echocardiography) [17].

### Statistical analysis

Using the formula proposed by Kelsey [18], we estimated, based on prior studies [19, 20], that 50% of the study cohort would achieve 80% adherence to every 4 week injections of benzathine Penicillin. We also expected a rate of recurrence of complications of 5% in those achieving less than 80% adherence from previous studies. Therefore, with a two sided alpha and 80% power, a sample size of 440 (220 per group including 10% allowance for losses to follow up), our study would detect atleast 8% difference in complication rate between participants with >80% adherence versus those with <80% adherence.

Univariate and bivariate analyses were conducted using frequencies and chi square tests for categorical variables, and means (standard deviation)/median (IQR) and Students t- test/ANOVA for continuous variables. Time to occurrence of morbidity or mortality outcomes were described using Kaplan-Meier curves. Differences between curves were described using log rank tests and hazard ratios (95% confidence intervals) were estimated using multivariate Cox proportional hazards models respectively. Covariates with *p* < 0.2 in the univariate (unadjusted) analysis or those with biological plausibility for outcomes were added into a multivariable model. Model goodness of fit was assessed using likelihood ratio tests (LRTs). We repeated all analyses separately by gender given the expected female predominance of RHD seen in our cohort. All data were managed using EPI data format 3.0 (Odensk, Denmark) [21] and were analyzed using STATA 12 (STATA Corporation, College Station, Texas, USA). A *p*-value <0.05 was considered statistically significant.

## Results

Baseline characteristics of the 449 study participation can be found in Table 1. There were twice as many female as male participants (300 female vs. 149 males). The mean age of enrollment was 31.4 years. As previously reported [7], initial presentation of RHD in our cohort occurred late in disease. At the time of presentation, 46% had heart failure of any stage, out of which 29% of patients were classified as NYHA stage 3 or higher. 9% had had a previous stroke, 4% had infective endocarditis, and 20% had atrial fibrillation. One hundred eighteen cases (26.3%) were lost to follow up and only seen on initial visit. Thus, outcomes data is determined from the remaining 331 patients.

**Table 1:** Baseline characteristics of study participants

Variable	Category	N (%)
Sex	Female	300 (66.8)
Age		
	<15	73 (16.3)
	16–30	150 (33.6)
	31–49	160 (35.6)
	>50	66 (14.7)
	Mean	31.4
Education		
	None	49 (10.1)
	Primary	215 (47.9)
	Secondary	131 (29.2)
	Vocational	16 (3.6)
	University	38 (8.5)
Occupation		
	None	115 (25.6)
	Student	206 (45.8)
	Small business	119 (26.5)
	Civil servant	9 (2.0)
NYHA Class		
	Class 1	228 (50.8)
	Class 2	91 (20.3)
	Class 3	110 (24.5)
	Class 4	20 (0.9)
Complications		
	Heart failure	208 (46.3)
	ARF	61 (13.6)
	Stroke	9 (2.0)
	Infect Endocarditis	18 (4.0)
	Atrial fibrillation	91 (20.3)
Echocardiography		
	LVIDd >55 mm	211 (46.9)
	LVIDs >40 mm	146 (32.5)
	Left atrial diameter >40 mm	364 (81.1)
	Ejection fraction <40	28 (6.2)
	Mitral regurgitation	304 (67.7)
	Mitral stenosis	131 (29.1)
	Aortic regurgitation	150 (33.4)
	Aortic stenosis	17 (3.8)
Laboratory		
	ASO titer >240	61 (13.6)
	C reactive protein >5	152(33.9)

*N* number, *NYHA* New York Heart Association, *ARF* acute rheumatic fever, *LVIDd* left ventricular internal dimension in diastole, *LVIDs* left ventricular internal dimension in systole, *ASO* anti-streptolysin-O

### Morbidity

**Fig. 1:**
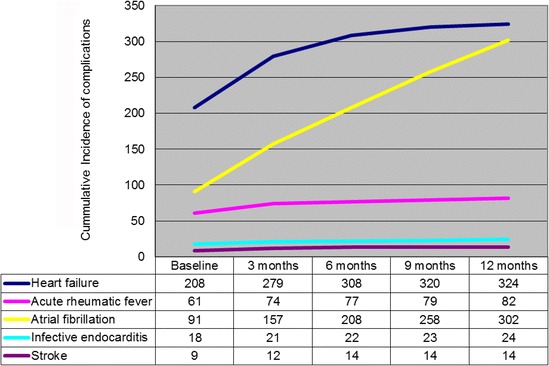
Morbidity During the First Year Following Initial RHD Presentation

Complications within 1-year of presentation were frequent and are presented in Fig. 1. Complications were most likely to occur within the first 3 months of presentation. By the end of the year, an additional 35% (116 patients) had developed heart failure, 6% (21 patients) had suffered an attack of recurrent acute rheumatic fever, 64% (211 patients) developed atrial fibrillation, 2% (six patients) developed infective endocarditis, and 2% (five patients) developed a stroke. New occurrence of heart failure was associated with penicillin adherence <80% (unadjusted OR = 3.3, CI 2.0–5.4, *p* = 0.001) and left ventricular end diastolic diameter greater than 55 mm (unadjusted OR 3.16, 95% CI 1.73–5.76, *p* = 0.001). Acute rheumatic fever was associated with younger age (<15 years, unadjusted OR = 0.92, 95% CI 0.88–0.95, *p* = 0.001) and poor penicillin adherence (unadjusted OR = 33.9 CI 7.58–151.3, *p* = 0.001).

### Mortality

**Fig. 2:**
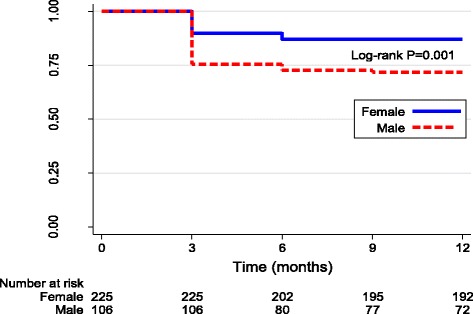
Kaplan- Meier curve for mortality within one year of initial presentation with rheumatic heart disease

There were 59 cardiac related deaths in this cohort with a 1-year mortality of 17.8%. The mean age of death was 29.4 years (+/− 15.5). Most deaths occurred within the first 3 months of presentation and males had higher risk of mortality compared to females (Fig. 2).

A univariate analysis of risk factors for mortality is found in Table 2. Patients were at higher risk of mortality if they were male (*p* = 0.001), had heart failure at presentation (*p* = 0.001), or had surrogate markers for heart failure, including left ventricular end diastolic dimension greater than 55 mm (*p* = 0.01), left ventricular peak systolic dimension greater than 40 mm (*p* = 0.01), ejection fraction <40% (*p* = 0.01). Markers of recent streptococcal infection or persistent inflammation including elevated ASO titer (*p* = 0.01) or elevated CRP (*p* = 0.001) during follow-up were also predictive of mortality. While severity of mitral stenosis, aortic stenosis, and aortic regurgitation were not predictive of death, severe mitral regurgitation was a risk factor for 1-year mortality (*p* = 0.001). Additionally, occurrence of infective endocarditis (0.001) or atrial fibrillation (0.007) during the study period also increased the risk of mortality.

**Table 2:** Univariate analysis of predictors of mortality stratified by gender

	Female		Male	
	HR (95% CI)	*P* value	HR (95% CI)	*P* value
Age (Ref: ≤15 years)				
16–30 year	1.47 (0.40–5.33)	0.561	0.53 (0.22–1.26)	0.153
31–49 years	0.96 (0.26–3.63)	0.957	0.57 (0.23–1.41)	0.226
>50 year	2.21 (0.59–8.32)	0.242	0.36 (0.047–2.86)	0.339
Education level (Ref: no education)				
Primary	1.26 (0.42–3.73)	0.682	1.60 (0.21–12.04)	0.650
Secondary	0.77 (0.22–2.62)	0.672	1.56 (0.20–12.00)	0.671
Post secondary	0.23 (0.03–2.04)	0.187	0.44 (0.03–7.04)	0.562
Occupation (Ref: unemployed)				
Student	0.60 (0.24–1.50)	0.274	1.09 (0.45–2.63)	0.845
Small business	0.41 (0.14–1.14)	0.086	0.32 (0.086–1.23)	0.099
Civil servant	**0.21 (0.06–0.75)**	**0.016**	0.504 (0.17–1.45)	0.206
Penicillin adherence (Ref: ≥80%)				
<80% adherence	**3.82 (1.55–9.38)**	**0.003**	**7.34 (2.56–21.11)**	**0.001**
NYHA at baseline (Ref: NYHA 1)				
Class 2	0.50 (0.17–1.50)	0.218	0.47 (0.17–1.29)	0.147
Class 3	0.97 (0.38–2.48)	0.947	0.62 (0.25–1.52)	0.305
Class 4	8.56 (2.48–29.52)	**0.001**	1.42 (0.32–6.18)	0.640
Mitral regurgitation (Ref: Mild MR)				
Moderate	2.86 (0.36–22.88)	0.322	0.43 (0.12–1.62)	0.215
Severe	8.38 (1.12–62.43)	**0.038**	1.47 (0.51–4.305)	0.740
Mitral stenosis (Ref: Mild MS)				
Moderate	0.51 (0.13–2.02)	0.334	0.37 (0.09–1.50)	0.165
Severe	0.41 (0.09–1.84)	0.244	0.28 (0.062–1.25)	0.097
Aortic regurgitation (Ref: Mild AR)				
Moderate	1.88 (0.60–5.92)	0.282	0.79 (0.25–2.509)	0.697
Severe	1.98 (0.38–10.20)	0.415	0.84 (0.25–2.87)	0.781
LVIDd >55 mm	1.90 (0.57–6.29)	0.290	1.95 (0.87–4.38)	0.105
LVIDs >45 mm	2.02 (0.48–8.49)	0.340	2.02 (0.77–5.28)	0.152
Ejection fraction >40%	0.50 (0.24–1.04)	0.060	0.60 (0.29–1.23)	0.167
Fractional shortening >20%	3.61 (1.09–11.93)	0.035	1.62 (0.49–5.36)	0.420
Pericardial effusion	0.26 (0.10–0.64)	0.004	0.82 (0.25–2.71)	0.750
Heart failure	12.32 (3.73–40.70)	0.001	11.07 (2.62–46.67)	0.001
Atrial fibrillation	1.87 (0.87–4.03)		2.06 (0.98–4.34)	0.056
Infective endocarditis	4.19 (1.45–12.08)	0.008	2.40 (0.73–7.93)	0.150
Acute rheumatic fever	1.09 (0.38–3.13)	0.870	1.42 (0.58–3.48)	0.440
Antistreptolysin O titers >240	1.14 (0.39–3.26)	0.810	1.54 (0.63–3.77)	0.340
C reactive protein >5 mg/L	2.78 (1.31–5.87)	0.007	1.78 (0.87–3.66)	0.110

Bolded results represent *p* < 0.05. Abbreviations as in Table 1

### Adherence to benzathine penicillin

**Fig. 3:**
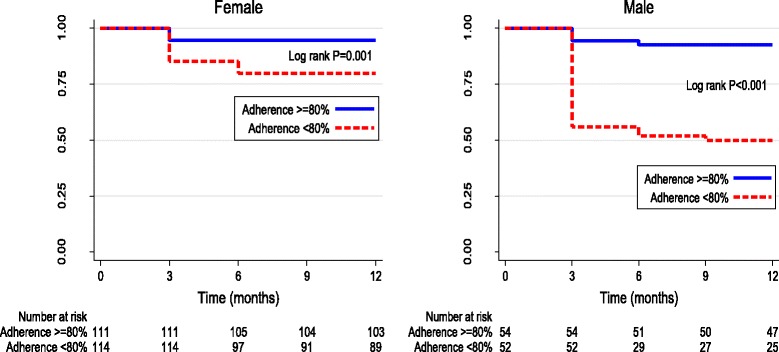
Kaplan Meir curves of participant survival stratified by benzathine penicillin adherence (<80% versus >80%)

All patients in this cohort were prescribed every-4-week injections of benzathine penicillin for streptococcal prophylaxis. The overall use of penicillin was 67.2%, with 57.5% of patients receiving at least 80% of their injections. Subjects who averaged less than 80% adherence had a significantly greater 1-year mortality compared to those who averaged greater than 80% adherence (31% vs. 9% mortality- for <80% adherence vs >80%adherence in the overall cohort, log-rank *p* < 0.001 for both men and women; Fig. 3).

Demographic information comparing those who were adherent to those who were not, separately by gender, is found in Table 3. Adherence to penicillin decreased with increasing age (71% of those <15 years compared to 22% of those >50 years, *p* < 0.001). Additional factors associated with poor compliance included no formal education (*p* = 0.001), and presence of stroke (0.04) or atrial fibrillation (*p* < 0.001). Elevated ASO titers and acute rheumatic fever at entry into the study showed borderline significance for poor penicillin compliance (*p* = 0.05 and; *p* = 0.06, respectively). Interestingly, there was no difference in adherence based on gender (*p* = 0.093), heart failure at presentation (*p* = 0.31), or any of the surrogate markers of heart failure (Table 3).

**Table 3:** Comparison of baseline characteristics of <80% versus ≥80% average adherence to benzathine penicillin

	Female			Male		
	<80%	≥80%	*P*-Value	<80%	≥80%	*P*-Value
Age						
≤15 years	31 (8.49)	85 (18.24)	<0.001	24 (17.91)	39 (18.57)	<0.001
16–30 year	66 (18.08)	191 (40.99)		52 (38.81)	91 (43.33)	
31–49 years	159 (43.56)	167 (35.84)		38 (28.36)	77 (36.67)	
>50 year	109 (29.86)	23 (4.94)		20 (14.93)	3 (1.43)	
Education level						
No education	86 (23.56)	24 (5.15)	<0.001	10 (7.46)	7 (3.33)	0.022
Primary	150 (41.10)	200 (42.92)		61 (45.52)	101 (48.10)	
Secondary	80 (21.92)	166 (35.62)		54 (40.30)	69 (32.86)	
Post secondary	49 (13.42)	76 (16.31)		9 (6.72)	33 (15.71)	
NYHA at baseline						
Class 1	191 (52.33)	241 (51.72)	0.823	53 (39.55)	81 (38.57)	0.833
Class 2	97 (26.58)	121 (25.97)		38 (28.36)	58 (27.62)	
Class 3	70 (19.18)	98 (21.03)		41 (30.60)	64 (30.48)	
Class 4	7 (1.92)	6 (1.29)		2 (1.49)	7 (3.33)	
Heart failure						
No	201 (55.07)	312 (66.95)	<0.001	56 (42.11)	120 (57.14)	0.007
Yes	164 (44.93)	154 (33.05)		77 (57.89)	90 (42.86)	
ARF at baseline						
No	341 (93.42)	386 (82.83)	<0.001	117 (87.31)	177 (84.29)	0.437
Yes	24 (6.58)	80 (17.17)		17 (12.69)	33 (15.71)	
Stroke						
No	349 (95.62)	465 (99.79)	<0.001	129 (96.27)	210 (100)	0.009
Yes	16 (4.38)	1 (0.21)		5 (3.73)	0 (0.0)	
Infective endocarditis						
No	352 (96.44)	455 (97.64)	0.305	121 (90.30)	210 (100)	<0.001
Yes	13 (3.56)	11 (2.36)		13 (9.70)	0 (0.0)	
Atrial fibrillation						
No	242 (66.30)	419 (89.91)	<0.001	102 (76.12)	176 (83.81)	0.077
Yes	123 (33.70)	47 (10.09)		32 (23.88)	34 (16.19)	
LVIDd						
<55 mm	208 (56.99)	282 (60.52)	0.305	56 (41.79)	109 (51.90)	0.067
≥55 mm	157 (43.01)	184 (39.48)		78 (58.21)	101 (48.10)	
LVIDs						
<40 mm	253 (69.32)	334 (71.67)	0.459	79 (58.96)	152 (72.38)	0.010
≥40 mm	112 (30.68)	132 (28.33		55 (41.04)	58 (27.62)	
Ejection fraction						
<40%	92 (25.48)	124 (26.61)	0.715	36 (26.87)	53 (25.24)	0.737
≥40%	269 (74.52)	342 (73.39)		98 (73.13)	157 (74.76)	
Left atrial diameter						
<40 mm	79 (21.64)	124 (26.61)	0.098	11 (8.21)	31 (14.76)	0.070
≥40 mm	286 (78.36)	342 (73.39)		123 (91.79)	179 (85.24)	
ASO						
<240	339 (94.17)	383 (82.90)	<0.001	117 (87.31)	181 (86.19)	0.765
≥240	21 (5.83)	79 (17.10)		17 (12.69)	29 (13.81)	
CRP						
<5 mg/L	227 (63.06)	335 (73.63)	0.001	87 (64.93)	129 (61.43)	0.513
≥5 mg/L	133 (36.94)	120 (26.37)		47 (35.07)	81 (38.57)	

Values shown are number (%)

### Multivariate analysis

In an effort to control for confounding variables, a multivariate analysis was conducted. At the univariate level (Table 2), male sex, heart failure at study entry, severe mitral regurgitation, left ventricular end diastolic dimension >55 mm, left ventricular end systolic dimension >40 mm, atrial fibrillation, infective endocarditis, elevated CRP, and benzathine penicillin adherence less than 80%, were significantly associated with increased mortality. After adjustment in the Cox proportional model, penicillin adherence <80% (HR = 3.81, 95% CI 1.92–7.63, *p* = 0.001), heart failure (HR = 8.36, CI 3.28–21.31, *p* = 0.001) and left ventricular diastolic diameter > 55 mm (HR = 1.93, CI 1.07–3.49, *p* = 0.02) were the only predictors of mortality (Table 4). Left ventricular diastolic diameter >55 mm and heart failure were not collinear in our model.

**Table 4:** Multivariate analysis of predictors of 1-year mortality

	HR 95% CI	*P* value
Penicillin adherence <80%	3.81 (1.92–7.63)	0.001
Sex (Male)	1.63 (0.96–2.77)	0.070
Heart failure at baseline	8.36 (3.28–21.31)	0.001
NYHA Class 2	0.42 (0.19–0.88)	0.020
NYHA Class 3	0.72 (0.37–1.38)	0.320
NYHA Class 4	2.16 (0.83–5.66)	0.110
Left ventricular diameter in diastole >55 mm	1.93 (1.07–3.49)	0.02
Pericardial effusion	0.5 (0.24–1.04)	0.06

## Discussion

Results of this first follow up study on outcomes of RHD in Uganda show a remarkably high mortality within 1-year of initial diagnosis of RHD, indicating that patients present for care at a very late stage of disease. The most important finding of our study is that even when mortality is driven by progressive heart failure, achieving greater than 80% adherence to penicillin significantly decreases mortality. To our knowledge, this is the first study to convincingly show a mortality benefit of optimal penicillin adherence in this population of patients with advanced disease who generally do not have access to cardiac surgery.

In this population, patients with less than 80% penicillin adherence to monthly injections of benzathine penicillin were much more likely to die than those who achieved more than 80% adherence, and this was true for both men and women. Other predictors of mortality included any degree of heart failure at baseline, dilated left ventricle, low ejection fraction, and advanced NYHA functional class. Our findings extend the results of the larger Global Registry of Rheumatic Heart Disease (REMEDY) study of over 3000 patients with RHD from low and middle income countries [10], of which our cohort was a subset. Mortality in the first year of REMEDY was 11%, somewhat lower than the 18% seen in the Ugandan cohort. This is not suprising considering the available resources for RHD care in Uganda at the time this cohort was recruited (2011–2013). In REMEDY, congestive heart failure, NYHA class, and prescription for penicillin for univariate predictors of mortality; although the association of penicillin prophylaxis with mortality did not persist in multivariable models. Penicillin adherence was not reported. Consistent with REMEDY, females had lower risk of mortality than males in our cohort.

Our study compliments the recently reported registry findings from the Northern Territory of Australia [22].

The patients in our study had more advanced disease, and, therefore, morbidity and mortality was significantly higher. Among 1149 patients diagnosed with RHD in the Australian cohort, age, gender, and indigenous status did not predict the development of heart failure; the effects of penicillin adherence or surrogate echocardiographic measures were not evaluated. This study did not evaluate the effect of penicillin adherence on mortality.

This study adds to previous findings that advanced valvular disease is common at first presentation [7, 23].

In the present study, acutely decompensated heart failure at baseline predicted mortality, but acute decompensation of heart failure and atrial fibrillation also occurred as the most common complications over the 12 months. This was probably due to progression of old lesions as well as occurrence of new heart failure in those who did not have signs at enrollment. Atrial fibrillation was explained by the large majority of participants having left atrial diameter greater than 40 mm. Interestingly, stroke rates remained low despite the high prevalence of atrial fibrillation. This could be explained by the fact most patients were prescribed Coumadin or an antiplatelet in those without facilities for monitoring the international normalized ratio (INR).

In the study by Bland and Jones [4], where 1000 cases of rheumatic heart disease were followed up for over 20 years, dilated left ventricle and heart failure at baseline accounted for over 80% mortality in the first 10 years. Although our follow up lasted only a year, our study seems to concur with their observation. Heart failure and dilated left ventricle at baseline were some of the independent predictors of mortality. Furthermore, the follow up by Bland et al. started in the pre penicillin era and only the last 5 years of the study had participants receiving penicillin [4].

More recent studies indicate that the prognosis of RHD is poor if left untreated—mortality occurs early with an average age of death of 26 years [20].

Gunther et al. [20] have reported that the annual mortality rate from RHD at a center in Ethiopia was 12.5%, about the same in our study where the annual mortality was 17.6%. The average age among participants who died in our study was 29.4 years, confirming the aggressive nature and poor prognosis of RHD in Uganda.

In the present study, poor adherence to penicillin was a major risk factor for death, but was also associated with complications such as atrial fibrillation, acute rheumatic fever and heart failure. Acute rheumatic fever recurrence was common and was predictably associated with poor penicillin adherence. Although younger people tended to be more adherent to penicillin, they were also more likely to suffer recurrence of disease. This is likely because children and adolescents are more likely to live in crowded conditions such as schools, a favorable environment for the spread of Group A streptococcal infection.

Intramuscular benzathine penicillin injection is the main stay of treatment among patients with chronic rheumatic heart disease. Penicillin, given either monthly or every 3 weeks reduces the rate of recurrence of acute rheumatic fever among patients with a history of rheumatic fever [24, 25], With good adherence to penicillin, the prognosis of RHD has been observed to improve, with more adherent patients experiencing less episodes of acute rheumatic fever. Tompkins et al. [5], in a 5 year follow of children with ARF and RHD showed that there was no mortality in the group that was fully adherent to penicillin. A small number of deaths occurred among patients who were non-adherent to prophylaxis or who had recurrent attacks of ARF and infective endocarditis [5].

Adequate penicillin adherence is generally defined as adherence level of 80% and above, calculated from the number of injections in a given period, divided by the number of injections expected in that period. Due to a number of factors including fear of painful injection, lack of access and lack of affordability, patients with RHD in Uganda have poor adherence to penicillin [19].

In the present study, adherence to penicillin was similarly low at 57.4%. Our study supports existing guidelines that recommend the consideration of lifelong penicillin prophylaxis among those with severe baseline RHD [26].

To our knowledge, ours is the first observational study to show a mortality benefit of >80% adherence compared to <80% adherence in a population with a high prevalence of advanced disease. Recurrent episodes of rheumatic fever may lead to severe decompensation and death among patients who have little reserve. This may be especially true for patients with severe valvular dysfunction who do not have access to cardiac surgery, and may even be true for older patients who are thought to be at low risk for rheumatic fever recurrences.

### Limitations

This study has several limitations. First, we acknowledge the relatively high rate of loss- to- follow- up (26%) which may have introduced bias into our study; however this is to be expected for studies conducted in very resource-limited settings and is in line with the REMEDY study (75% with 2-year follow-up visit or telephone interview). Furthermore, we might expect that those lost to follow-up were more likely to die and were less likely to be optimally adherent to penicillin, thus our results may be biased towards lower estimates of mortality and lower estimated effectiveness of penicillin.

Another important limitation of our study is the inability to completely adjust for confounding in our multivariable analysis of the effect of penicillin adherence on mortality. It should be noted that the <80% and >80% adherence groups had significant differences in baseline characteristics, including severity of illness. This is to be expected given that adherence behaviors likely predated the enrollment into our RHD registry and may have influenced the baseline severity of disease. Although we attempted to adjust for these differences in our multivariable analysis, the possibility of residual confounding persists. Only a randomized trial of penicillin prophylaxis in this population could overcome this limitation.

Finally, we determined adherence based on a benzathine penicillin card marked by the attending health worker whenever an injection was given. A more sure method would be to determine penicillin levels to determine adherence. Finally, most events we followed our patients for, occurred between the three monthly visits making it impossible to know the precise time of occurrence of events. We tried to overcome this by using interval assumption during survival analysis.

## Conclusion

This study suggests that there is high mortality among RHD patients in Uganda and that poor adherence to penicillin and heart failure from advanced valvular disease predict mortality. Future studies should test interventions to improve adherence among patients with advanced disease who are at the highest risk of mortality.

## Abbreviations

ANOVA: Analysis of variance
ASO: Antistreptolysin O titers
CRP: C-reactive protein
GEE: Generalized estimating equations
NIH: National Institutes of Health
RF: Rheumatic fever
RHD: Rheumatic heart disease
WHO: World Health Organization
